# An analysis of the trend towards universal health coverage and access to healthcare in Morocco

**DOI:** 10.1186/s13561-023-00477-0

**Published:** 2024-01-20

**Authors:** Tarek Drissi Bouzaidi, Aziz Ragbi

**Affiliations:** Faculty of Law, Economic and Social Sciences, Mohammed V University, Rabat, Morocco

**Keywords:** UHC, Health care access, ONDH, Morocco

## Abstract

**Objective:**

We aim in this study to investigate the association between access to health care services and various components of universal health coverage in Morocco, controlling for socioeconomic, demographic, and cultural factors.

**Data and methods:**

The study employed a logistic regression method to model the relationship between access to health care as binary outcome variable and health coverage, using the longitudinal data collected from the Household Panel Survey of the National Observatory of Human Development (ONDH) spanning the period from 2013 to 2019.

**Results:**

The study reveals a significant association between access to health care services and having medical coverage taking into consideration socioeconomic and demographic characteristics as the main determinants of access to health care services.

**Conclusion:**

The study investigates the impact of demographic and socioeconomic factors on medical care utilization. The econometric model reveals that individuals with medical coverage, particularly through AMO and RAMED, are more likely to seek health care services, emphasizing the positive influence of universal health coverage. Additionally, demographic and socioeconomic characteristics such as gender, education, employment, and living environment significantly affect health care-seeking behavior. Urban residents, women, and those with higher standards of living are more inclined to access health care services.

## Introduction

The advent of the health crisis linked to the Covid-19 pandemic, whose consequences are having a severe impact at the national and international levels, has once again demonstrated the urgency for countries to work on strengthening their health systems by accelerating efforts to achieve Universal Health Coverage (UHC). Undoubtedly, ensuring universal healthcare coverage is essential for enhancing a nation's population well-being, fostering investment in human capital, and laying a strong foundation for all-inclusive and sustainable economic growth and progress. It presents an opportunity to provide support to individuals, enabling them to realize their full potential and achieve their ambitions. The 2030 Agenda for Sustainable Development that includes 17 Sustainable Development Goals (SDGs) adopted in September 2015 by the United Nations General Assembly, with the third goal of the agenda focusing on health – good health and well-being. Realizing universal health coverage, which entails access to affordable and quality essential health care services, safe, effective, and affordable essential drugs, and vaccines for all (irrespective of ability-to-pay), with financial risk protection [1–4]. According to the Global Monitoring Report of 2017, approximately 50% of the world's population still does not have access to necessary health care services, and every year, nearly 100 million people fall into extreme poverty due to healthcare-related expenses. The 2019 Global Monitoring Report on financial protection in health emphasizes the financial aspect of achieving universal health coverage, particularly the burden of out-of-pocket expenses. The report investigates the number of people worldwide who are forced into poverty due to Out-Of-Pocket (OPP) healthcare payments and how much of their consumption or income is taken up by these expenses, known as “catastrophic expenditures” when they exceed 10% or 25%. The report reveals that globally, about 925 million people spend more than 10% of their household income on healthcare, and over 200 million spend more than 25%. The implementation of UHC is part of a framework carried by international institutions that leads to a plurality of UHC models [5, 6]. This framework is the subject of many questions, that show its problematic effects on low- and middle-income countries [7]. Indeed, each model highlights one aspect, leaving another in the dark. Hence, there is no «*one best way*» to construct a UHC, and no study can prove that one model is better than the other.

Like many countries, Morocco had launched universal health coverage with insurance, contributory and assistance dimensions, supported by a Compulsory Health Insurance (AMO) since 2005 for public and private employees, and accompanied by an ambitious scheme to ensure access to needed health care services for the most disadvantaged: The RAMED (Medical Assistance Regimes for the Economically Disadvantaged) was initiated in 2008 and implemented in 2011, then extended to the entire population in 2017. This program is designed for disadvantaged or poor populations and allows them access to public health services, solely in the public sector. This was a major social reform that was imposed on the government in regard to the demographic and epidemiological transitions, the rising health costs and the strong social demands since the late 1990s.

While Morocco has made great efforts to improve the health coverage of its population, the general feeling is that the negative effects outweigh the positive ones. The population has reacted vigorously and severely to the various shortcomings and dysfunctions, particularly in terms of disparities in resources between and within regions, inequity in access to health services, and less-than-optimal governance expressed in a supply of care that is inadequate to health demand and needs [8].

In this article, we aim to develop the relationship between universal health coverage with its different components and the access to health care services in Morocco, basing on longitudinal data from the Household Panel Survey of the National Observatory of Human Development (*ONDH*) over the period from 2013 to 2019. The findings can enhance the existing literature on the extent of health insurance coverage and access to healthcare services, and help formulate several recommendations on this matter.

## Background

As early as the 1960s, Kenneth Arrow showed that health economics was gaining the upper hand over medical economics [9]. The awareness in the medical field of the scarcity of resources (especially financial), and the need to optimize the performance of the system has led public decision-makers to invent new ideas and strategies. In his turn, *Grossman* inspired by the human capital theory, presents health services as intermediate goods and gives the example of healthy nutrition and physical activities, which enable a human to obtain a physical, mental and social well-being [10].

The earliest research examining the influence of insurance coverage on access to health care services can be traced back to the 20th century, specifically to the works of Arrow and Anderson [9, 11]. These studies, along with subsequent research, have demonstrated that health insurance has a positive impact on the utilization of health services, establishing a causal relationship between the two factors. Indeed, the provision of partial or full coverage of healthcare costs through insurance enables members to access a wide range of medical treatments, including the more costly ones. This largely accounts for the direct and positive correlation observed between health insurance coverage and access to healthcare services [12].

The first and only randomized experiment to examine the effects of health insurance on beneficiaries' health was conducted between 1974 and 1982 in the United States, called the RAND Experiment *Healt*h *Insurance Experiment* [13]. This experiment studied around 4000 people in 2000 households. Some families were randomly assigned to a free health plan, while others received one of several plans requiring variable copay (10%, 20%, 40%). The study found that those assigned to a cost-sharing plan sought less treatment than those with full coverage. The main purpose of health insurance is to improve access to health care and reduce individual out-of-pocket expenses, especially for people with limited income (Tables 1, 2 and 3).

**Table 1:** Medical coverage and demographic and socio-economic characteristics

**Characteristics**	**AMO** ***N* = 35,135**	**RAMED** ***N* = 47,045**	**Without** ***N* = 58,887**	**Overall** ***N* = 141,067**	***P*-value**
***Gender***					< 0.001
Male	17,634 (25%)	22,422 (32%)	30,009 (43%)	70,065 (100%)	
Female	17,501 (24%)	24,622 (35%)	28,878 (41%)	71,001 (100%)	
***Age_groups***					< 0.001
[0,15]	9,075 (26%)	13,511 (38%)	12,948 (36%)	35,534 (100%)	
[15,60]	20,919 (24%)	27,106 (31%)	39,805 (45%)	87,830 (100%)	
[60,99]	5,141 (29%)	6,428 (36%)	6,134 (35%)	17,703 (100%)	
***Residence***					< 0.001
Urban	27,365 (36%)	20,920 (27%)	28,397 (37%)	76,682 (100%)	
Rural	7,770 (12%)	26,125 (41%)	30,490 (47%)	64,385 (100%)	
***Marital status***					< 0.001
Married	17,626 (29%)	21,841 (36%)	21,466 (35%)	60,933 (100%)	
Single	16,120 (22%)	21,961 (31%)	33,723 (47%)	71,804 (100%)	
Other	1,389 (17%)	3,243 (39%)	3,698 (44%)	8,330 (100%)	
***Education***					< 0.001
Illiterate	8,806 (17%)	20,578 (40%)	21,684 (42%)	51,068 (100%)	
Elementary school	9,049 (22%)	14,990 (36%)	17,040 (41%)	41,079 (100%)	
Middle and high school	11,790 (31%)	10,327 (27%)	16,200 (42%)	38,317 (100%)	
College	5,490 (52%)	1,150 (11%)	3,963 (37%)	10,603 (100%)	
***Employment***					< 0.001
Yes	9,544 (29%)	9,365 (29%)	13,479 (42%)	32,388 (100%)	
No	21,390 (25%)	29,534 (34%)	35,249 (41%)	86,173 (100%)	
***Household size***					< 0.001
Without	14 (18%)	15 (19%)	49 (63%)	78 (100%)	
[1-4]	15,776 (29%)	17,149 (31%)	21,739 (40%)	54,664 (100%)	
[5-8]	18,168 (24%)	26,523 (35%)	31,746 (42%)	76,437 (100%)	
[9 et PLUS]	1,177 (12%)	3,358 (34%)	5,353 (54%)	9,888 (100%)	
***Medical consultation***					< 0.001
Yes	4,195 (30%)	5,086 (37%)	4,537 (33%)	13,818 (100%)	
No	1,001 (19%)	1,934 (37%)	2,321 (44%)	5,256 (100%)	
***Prenatal consultation***					< 0.001
Yes	4,713 (28%)	6,051 (36%)	5,992 (36%)	16,756 (100%)	
No	231 (11%)	996 (46%)	943 (43%)	2,170 (100%)	
***Delivery assisted by a specialist***					< 0.001
Yes	4,733 (28%)	6,160 (36%)	5,991 (35%)	16,884 (100%)	
No	211 (10%)	887 (43%)	946 (46%)	2,044 (100%)	
***Secteur consulted***					< 0.001
Private	2,719 (38%)	2,072 (29%)	2,402 (33%)	7,193 (100%)	
Public	1,445 (22%)	2,979 (46%)	2,105 (32%)	6,529 (100%)	
Other	19 (22%)	35 (40%)	34 (39%)	88 (100%)	
***Household expenses (medical care)***					< 0.001
* Median (IQR)*	1,217 (0, 4,260)	720 (0, 2,786)	913 (0, 3,154)	913 (0, 3,209)	
Range	0, 327,699	0, 115,636	0, 249,217	0, 327,699	
* Mean (SD)*	3,855 (9,258)	2,422 (5,471)	2,816 (6,449)	2,946 (7,011)	
***Distance from your home to the clinic or health center***					< 0.001
* Median (IQR)*	900 (500, 2,000)	1,500 (600, 5,000)	1,200 (500, 5,000)	1,000 (500, 4,000)	
Range	0, 90,000	0, 95,000	0, 99,999	0, 99,999	
* Mean (sd)*	2,216 (5,808)	4,213 (8,031)	4,108 (8,026)	3,672 (7,583)	
***Health status***					< 0.001
Chronic diseases	3,685 (28%)	4,976 (38%)	4,393 (34%)	13,054 (100%)	
Healthy	31,450 (25%)	42,069 (33%)	54,494 (43%)	128,013 (100%)	
***Wealth Quintile***					< 0.001
1^st^ quintile	2,140 (7.6%)	12,862 (46%)	13,001 (46%)	28,003 (100%)	
2^nd^ quintile	5,359 (15%)	14,282 (41%)	15,584 (44%)	35,225 (100%)	
3^rd^ quintile	10,172 (26%)	12,255 (31%)	16,575 (42%)	39,002 (100%)	
4^th^ quintile	17,464 (45%)	7,646 (20%)	13,726 (35%)	38,836 (100%)	
***Region***					< 0.001
RABAT_SALE_KENETRA	4,267 (28%)	5,240 (34%)	5,977 (39%)	15,484 (100%)	
CASA_SETTAT	6,015 (32%)	4,305 (23%)	8,578 (45%)	18,898 (100%)	
BENI_MELAL_KHNIF	2,271 (19%)	4,084 (34%)	5,712 (47%)	12,067 (100%)	
DAKH_OUED_DAHAB	1,015 (49%)	441 (21%)	610 (30%)	2,066 (100%)	
DERAA_TAFIL	3,175 (26%)	4,331 (35%)	4,816 (39%)	12,322 (100%)	
FES_MEKNES	3,012 (21%)	5,745 (40%)	5,570 (39%)	14,327 (100%)	
GUELM_OUED_NONE	3,093 (30%)	4,352 (42%)	2,996 (29%)	10,441 (100%)	
LAAYOU_SAKIA_HAM	2,047 (39%)	1,886 (36%)	1,354 (26%)	5,287 (100%)	
MARAKECH_SAFI	3,014 (19%)	5,322 (34%)	7,531 (47%)	15,867 (100%)	
ORIENATAL	1,804 (17%)	5,360 (50%)	3,623 (34%)	10,787 (100%)	
SOUS_MASSA	2,806 (27%)	2,494 (24%)	5,031 (49%)	10,331 (100%)	
TANG_TET_HOUSEIM	2,598 (20%)	3,480 (26%)	7,083 (54%)	13,161 (100%)

**Table 2:** Access to care and demographic and socio-economic characteristics

**Characteristics**	***Yes*** ***N* = *13,818***	***No*** ***N* = *5,2561***	***Overall*** ***N* = *19,074***	***p-value***
***Gender***				< 0.001
Male	5,891 (70%)	2,478 (30%)	8,369 (100%)	
Female	7,927 (74%)	2,778 (26%)	10,705 (100%)	
***Age_groups***				< 0.001
[0,15]	1,953 (70%)	819 (30%)	2,772 (100%)	
[15,60]	6,643 (71%)	2,729 (29%)	9,372 (100%)	
[60,99]	5,222 (75%)	1,708 (25%)	6,930 (100%)	
***Residence***				< 0.001
Urban	8,861 (78%)	2,557 (22%)	11,418 (100%)	
Rural	4,957 (65%)	2,699 (35%)	7,656 (100%)	
***Marital status***				< 0.001
Married	7,803 (74%)	2,801 (26%)	10,604 (100%)	
Single	3,594 (67%)	1,733 (33%)	5,327 (100%)	
Other	2,421 (77%)	722 (23%)	3,143 (100%)	
***Education***				< 0.001
Illiterate	7,637 (72%)	3,000 (28%)	10,637 (100%)	
Elementary school	3,134 (72%)	1,200 (28%)	4,334 (100%)	
Middle and high school	2,362 (74%)	834 (26%)	3,196 (100%)	
College	685 (76%)	222 (24%)	907 (100%)	
***Employment***				0.1
Yes	2,421 (75%)	826 (25%)	3,247 (100%)	
No	9,830 (73%)	3,691 (27%)	13,521 (100%)	
***Household size***				< 0.001
Without	11 (92%)	1 (8.3%)	12 (100%)	
[1-4]	6,809 (74%)	2,337 (26%)	9,146 (100%)	
[5-8]	6,366 (71%)	2,601 (29%)	8,967 (100%)	
[9 et PLUS]	632 (67%)	317 (33%)	949 (100%)	
***Secteur consulted***				
Private	7,190 (100%)	0 (0%)	7,190 (100%)	
Public	6,525 (100%)	0 (0%)	6,525 (100%)	
Other	88 (100%)	0 (0%)	88 (100%)	
***Household expenses (medical care)***				< 0.001
* Median* *(IQR)*	3,652 (1,826, 7,200)	1,577 (368, 3,496)	3,043 (1,337, 6,176)	
Range	0, 327,699	0, 249,217	0, 327,699	
* Mean (SD)*	6,519 (10,548)	3,220 (7,176)	5,669 (9,897)	
***Distance from your home to the clinic or health center***				< 0.001
* Median (IQR)*	1,000 (500, 3,000)	1,080 (500, 4,000)	1,000 (500, 3,000)	
Range	0, 97,000	0, 95,000	0, 97,000	
* Mean (sd)*	2,986 (7,042)	3,933 (7,739)	3,246 (7,253)	
***Health status***				< 0.001
Chronic diseases	10,126 (78%)	2,928 (22%)	13,054 (100%)	
Healthy	3,692 (61%)	2,328 (39%)	6,020 (100%)	
***Health coverge satatus***				< 0.001
AMO	4,537 (66%)	2,321 (34%)	6,858 (100%)	
RAMED	5,086 (72%)	1,934 (28%)	7,020 (100%)	
Without	4,195 (81%)	1,001 (19%)	5,196 (100%)	
***Wealth Quintile***				< 0.001
1^st^ quintile	1,311 (58%)	941 (42%)	2,252 (100%)	
2^nd^ quintile	2,611 (65%)	1,390 (35%)	4,001 (100%)	
3^rd^ quintile	3,899 (73%)	1,461 (27%)	5,360 (100%)	
4^th^ quintile	5,997 (80%)	1,464 (20%)	7,461 (100%)	
***Region***				< 0.001
RABAT_SALE_KENETRA	1,757 (80%)	437 (20%)	2,194 (100%)	
CASA_SETTAT	2,512 (81%)	581 (19%)	3,093 (100%)	
BENI_MELAL_KHNIF	936 (59%)	648 (41%)	1,584 (100%)	
DAKHLA_OUED_DAHAB	210 (85%)	37 (15%)	247 (100%)	
DERAA_TAFIL	1,005 (69%)	456 (31%)	1,461 (100%)	
FES_MEKNES	1,088 (68%)	512 (32%)	1,600 (100%)	
GUELMIM_OUED_NONE	1,141 (83%)	232 (17%)	1,373 (100%)	
LAAYOUNE_SAKIA_HAMRA	409 (79%)	108 (21%)	517 (100%)	
MARAKECH_SAFI	1,746 (77%)	523 (23%)	2,269 (100%)	
ORIENATAL	897 (65%)	477 (35%)	1,374 (100%)	
SOUS_MASSA	1,062 (70%)	456 (30%)	1,518 (100%)	
TANG_TET_HOUSEIMA	1,053 (57%)	788 (43%)	1,841 (100%)

**Table 3:** Regression model

*Characteristics*	*Wave_2017*			*Wave_2019*		
	***OR***	***95% CI***	***P-value***	***OR***	***95% CI***	***P-value***
***Health coverge satatus***						
RAMED ***vs*** without	1.24	1.10, 1.40	< 0.001	1.32	1.17, 1.49	< 0.001
AMO ***vs*** without	1.54	1.32, 1.80	< 0.001	1.34	1.17, 1.54	< 0.001
***Health status***						
Chronic diseases vs Healthy	2.21	1.95, 2.49	< 0.001	2.2	1.95, 2.47	< 0.001
***Age_groups***						
[15,60] vs [0,15]	0.9	0.72, 1.13	0.4	0.93	0.76, 1.14	0.5
[60,99] vs [0,15]	1.12	0.88, 1.43	0.3	0.9	0.72, 1.13	0.4
***Gender***						
Femelle vs male	1.37	1.21, 1.55	< 0.001	1.25	1.11, 1.40	< 0.001
***Residence***						
Urban vs Rural	1.39	1.23, 1.58	< 0.001	1.08	0.96, 1.22	0.2
***Education***						
Elementary vs Illiterate	1.21	1.04, 1.42	0.016	1.1	0.96, 1.27	0.2
Middle and high vs Illiterate	1	0.85, 1.17	> 0.9	1.09	0.93, 1.28	0.3
SUPERIEUR vs Illiterate	0.8	0.60, 1.07	0.13	1.02	0.80, 1.30	0.9
***Employment***						
Yes vs No	1.32	1.12, 1.55	< 0.001	1.21	1.05, 1.40	0.01
***Household size***	1.07	1.04, 1.10	< 0.001	1.08	1.05, 1.11	< 0.001
***Distance from your home to the clinic or health center***						
	1	1.00, 1.00	0.5	1	1.00, 1.00	0.8
***Wealth Quintile***						
2^nd^ quintile vs 1^st^ quintile	1.36	1.15, 1.61	< 0.001	1.45	1.23, 1.71	< 0.001
3^rd^ quintile vs 1^st^ quintile	2.03	1.69, 2.45	< 0.001	1.87	1.57, 2.23	< 0.001
4^th^ quintile vs 1^st^ quintile	3.01	2.44, 3.71	< 0.001	2.47	2.04, 3.01	< 0.001
***Region***						
RABAT_SALE_KENETRA	—	—		—	—	
CASA_SETTAT	0.81	0.65, 1.01	0.064	1.27	1.03, 1.59	0.029
BENI_MELAL_KHNIF	0.37	0.29, 0.46	< 0.001	0.47	0.37, 0.60	< 0.001
DAKHLA_OUED_DAHAB	0.53	0.28, 1.06	0.057	1.11	0.69, 1.89	0.7
DERAA_TAFIL	0.67	0.52, 0.86	0.002	1	0.78, 1.28	> 0.9
FES_MEKNES	0.72	0.56, 0.92	0.01	0.46	0.37, 0.57	< 0.001
GUELMIM_OUED_NONE	1.58	1.16, 2.16	0.004	0.99	0.77, 1.27	> 0.9
LAAYOUN_SAKIA_HAMRA	2.8	1.55, 5.61	0.002	0.4	0.30, 0.54	< 0.001
MARAKECH_SAFI	0.87	0.69, 1.10	0.2	1.19	0.96, 1.49	0.11
ORIENATAL	0.46	0.35, 0.59	< 0.001	0.59	0.47, 0.74	< 0.001
SOUS_MASSA	2.28	1.63, 3.21	< 0.001	0.45	0.36, 0.55	< 0.001
TANG_TET_HOUSEIMA	0.36	0.28, 0.45	< 0.001	0.54	0.43, 0.67	< 0.001

*OR* Odds Ratio, *CI* Confidence Interval

Previous studies on the use of health care services have shown that health insurance positively influences the use of health care services. Indeed, having insurance leads to more financing options, which has an impact on the insured’s choice health care provider [14].

Economic theory [15] and a large body of literature confirm the relationship between insurance coverage and access to care [16, 17] and satisfaction with the experience of care [18, 19]. Five main areas are considered in assessing the performance of health insurance policies: quality, accessibility, efficiency, continuity and equity [20]**.** Both the private and public sectors offer medical insurance coverage options; Medicare and Medicaid in the United States are a well-known example of a federal welfare affiliate.

Any improvement in access to care and insurance coverage could also improve the quality of life of citizens. Better insurance coverage would better support three goals: developing reliable sources of care for every individual, expanding access to medical, dental and vision care, and improving patient satisfaction in each area of care [21]. Previous studies have also found a positive relationship between the expansion of health insurance and increased patient satisfaction and quality of care [22].

The level of utilization of both curative and preventive health services can be related to the standard of living of households. Indeed, health systems often offer more services at a higher quality to the richest, while the poorest, who need them the most, cannot obtain them [23]. Inequalities in access to health care are also associated with the existence of significant income gradients, educational attainment, and overall social status [24]. Determinants such as gender, culture, education, employment, income, and place of residence are all closely related to access to health care [25].

“In all cases, people in disadvantaged socioeconomic groups tend to have higher rates of morbidity, disability, and mortality, use fewer preventive services and specialty care than would be expected based on their needs. They also tend to pay a larger share of their income to purchase certain health goods and services.” [26].

## Materials and methods

Most studies examining the connection between health insurance and health care utilization typically rely on survey data and utilize multivariate logistic regression analysis, especially when the dependent variable involves binomial or multinomial categorical outcomes. In the broader landscape of research, it is noteworthy that the study under consideration, which investigates the impact of demographic and socioeconomic factors on healthcare utilization, employs a similar method. Logistic regression is chosen to analyze longitudinal data from the Household Panel Survey of the National Observatory of Human Development (ONDH) spanning 2013 to 2019. This statistical approach allows for a comprehensive exploration of the relationships between health coverage, demographic characteristics, and socioeconomic factors in influencing the probability of seeking medical care. It is essential to acknowledge, as highlighted by Hadley [27] in a review of studies published between 1991 and 2001, that the majority found a positive correlation between health insurance coverage and recovery from various health conditions. However, the cautionary note raised in the review, emphasizing the potential limitations of causal interpretation due to unobserved heterogeneity and reverse causality in research designs, underscores the complexities inherent in drawing conclusive causal relationships in this domain.

## Top of form

### Data

Several public organizations and institutional entities in Morocco have been interested in the design, collection, exploitation and dissemination of data to assess the well-being of individuals by describing their socio-demographic, socio-economic and socio-cultural situation in order to evaluate the progress made in improving the living standards of the population. To this end, various cross-sectional and longitudinal (panel) surveys have been carried out, in particular the Household Panel Surveys (HPS) promoted by the National Observatory of Human Development (ONDH), the surveys on living standards carried out by the High Commission for Planning (HCP), the repeated demographic surveys, etc.

The baseline survey (Wave-1 of Panel I) conducted in 2012 with a sample of 8,000 households, was repeated in 2013, 2015, 2017 and 2019. In order to ensure regional representativeness of the human development results, the survey was expanded in 2017 and 2019 to include an additional 8,000 households, bringing the initial sample to 16,000 households. It should be noted that the morbidity and health care utilization questions cover the four-week reference period prior to the date of the interviewers' visit to the sample household, for more details http://www.ondh.ma/fr/enquete-panel-de-menage.

This paper uses this database with waves (2013 to 2019) to examine the relationship between medical coverage, specifically the two regimes of AMO and RAMED and health care access, while controlling for socioeconomic, demographic and cultural factors.

### Measures

#### Health insurance coverage

**Fig. 1:**
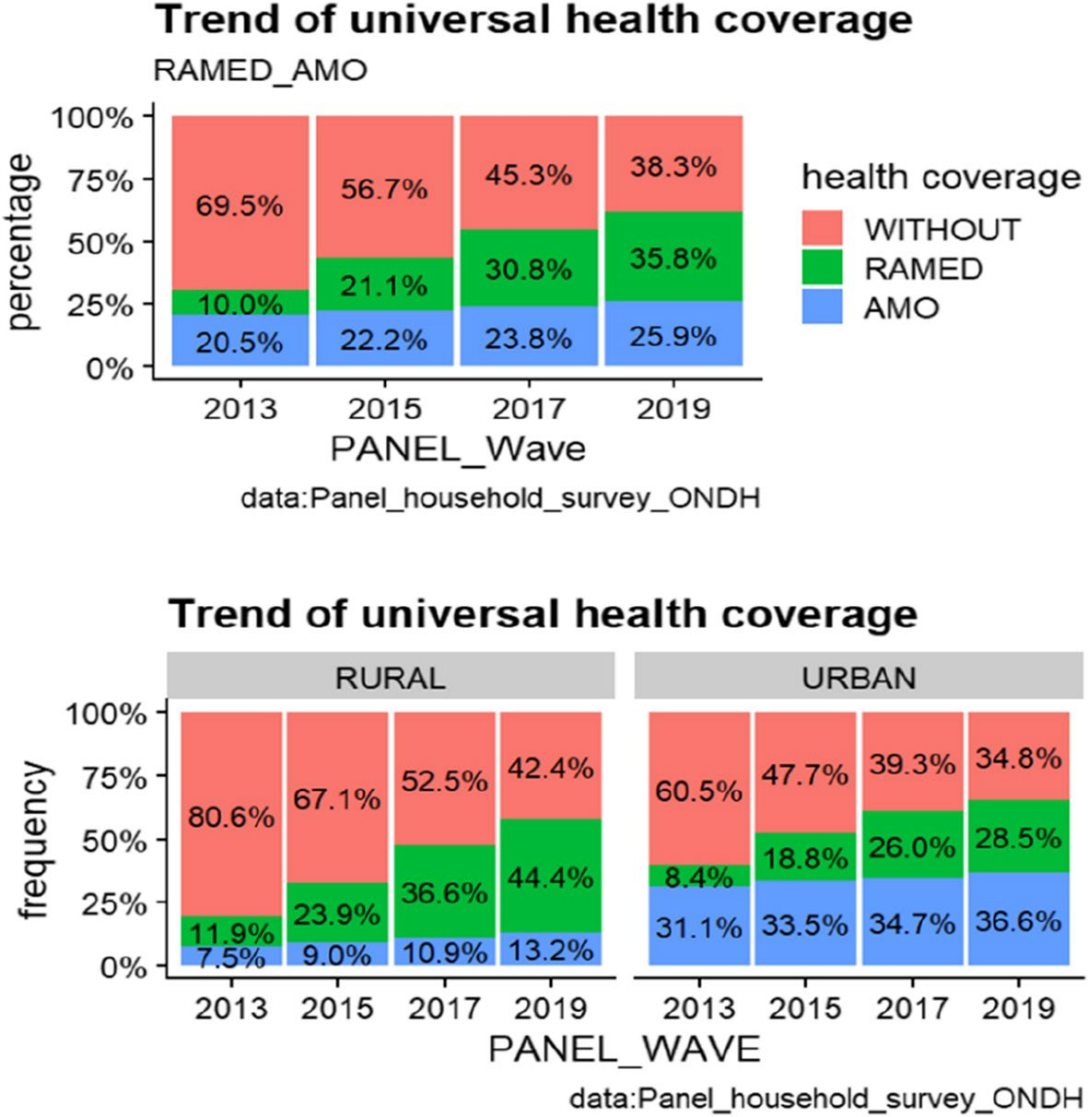
Trend of UHC

**Fig. 2:**
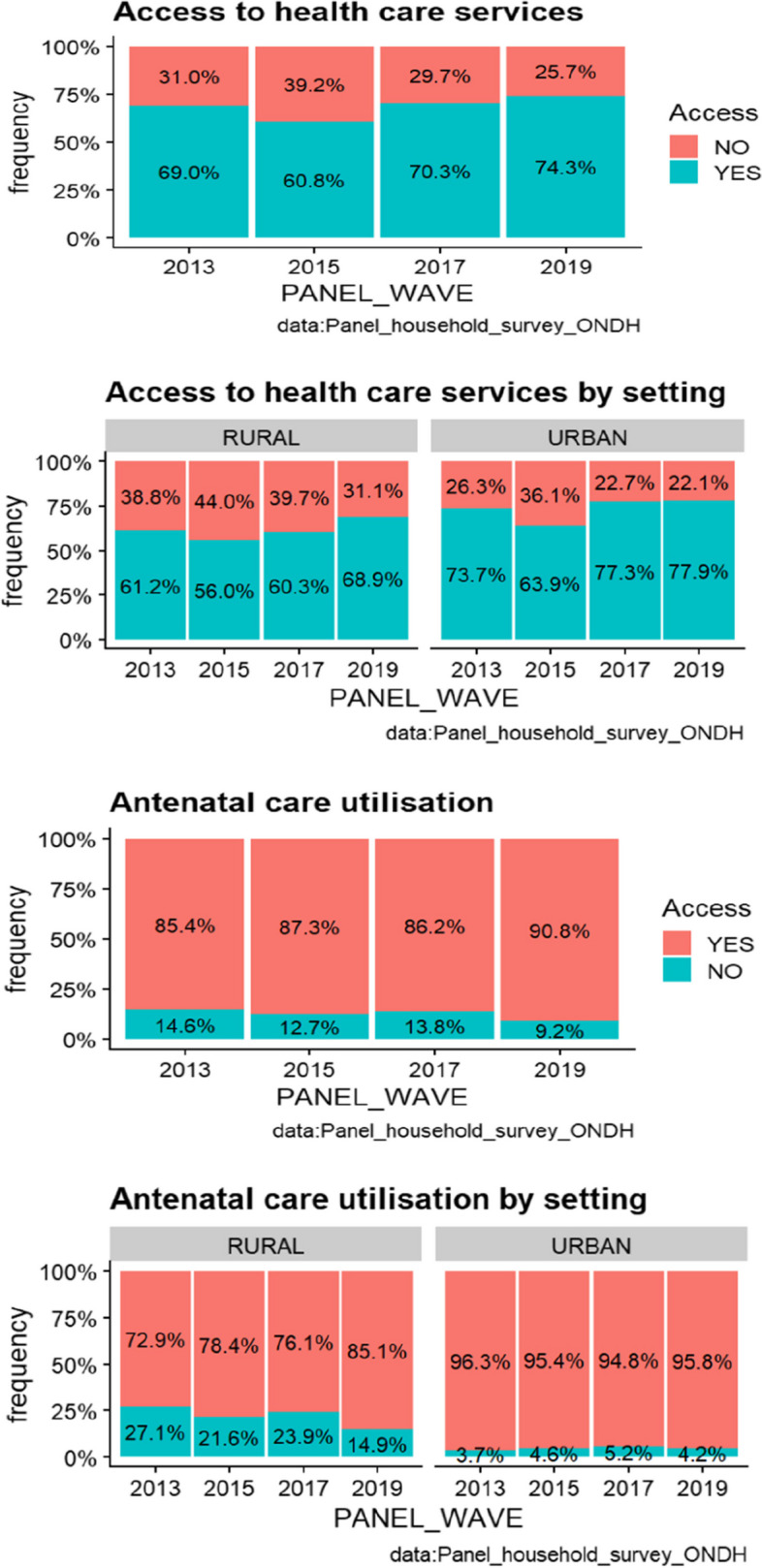
Utilization of health care

**Fig. 3:**
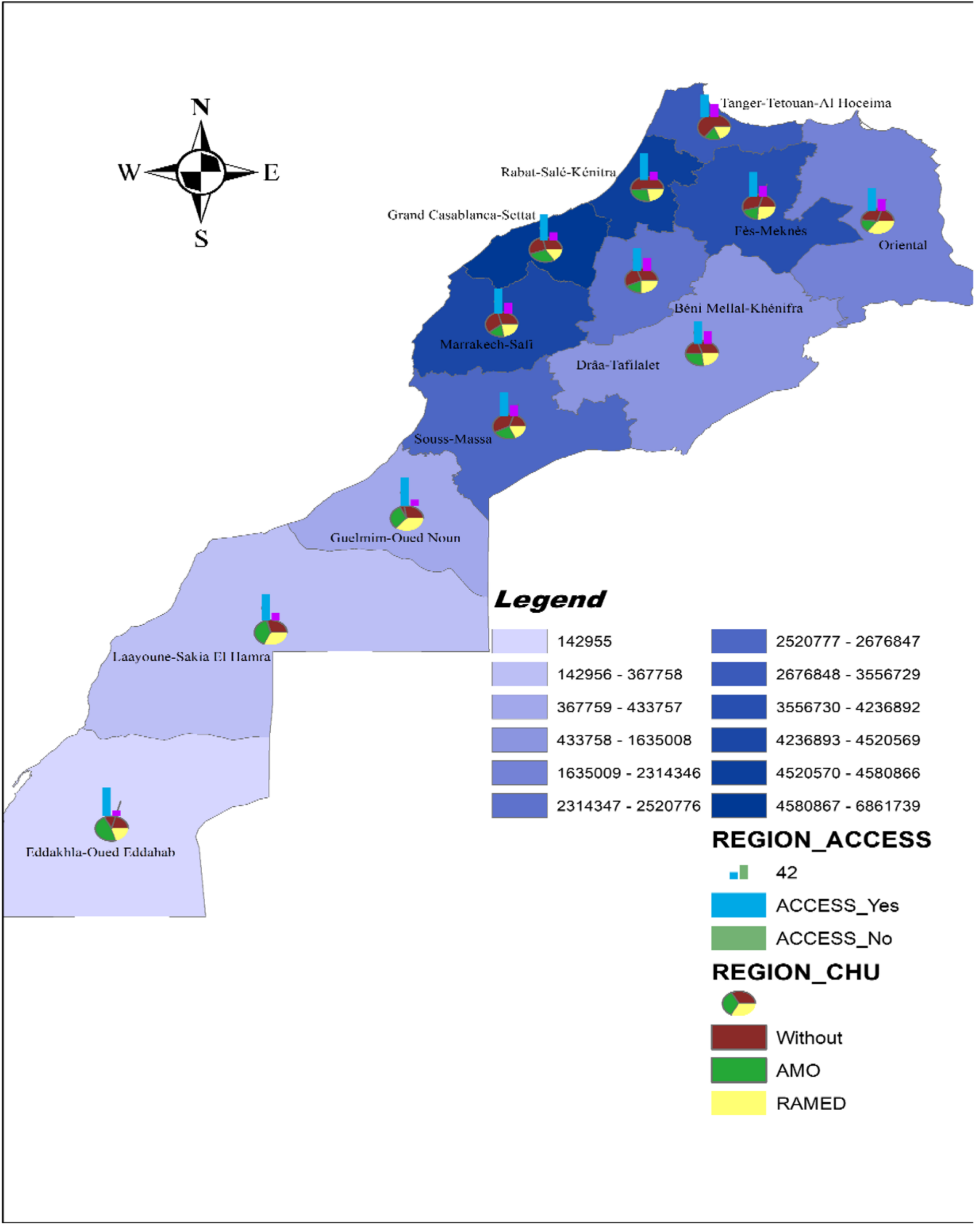
Geographical disparities in terms of care access, health coverage status and population

The advent of the two AMO and *RAM*ED regimes was a real social evolution that translates the Moroccan State commitment to ensure social cohesion and fight against vulnerability and precariousness. Indeed, the health coverage of the population has progressed significantly between 2013 and 2019, but is still far from reaching the principle of universality. Thus, the rate of coverage by health insurance, all types combined (AMO and RAMED), reached 61.7% in 2019, against 30.5% in 2013, an improvement of 31.2%. In this way, health insurance covers almost 53.4% of the urban population and 46.6% of the rural population (Figs. 1, 2 and 3).

An analysis of the structure of health coverage by type of insurance shows that the share of RAMED has improved significantly between 2013 and 2019, rising nationally from 10% in 2013 to 35.8% in 2019. This improvement is particularly noticeable in rural areas where RAMED coverage increased from 11.9% to 44.4% compared to 8.4% to 28.5% in urban areas between 2013 and 2019.

#### Access to care

In terms of medical consultation, the consultation rate, after recording a decline from 69% in 2013 to 60.8% in 2015, increased in 2017 and 2019 to reach 70.3% and 74.3% successively. As for the prenatal consultation rate covering the last pregnancy, it rose from 85.4% in 2013 to 90.8% in 2019, an improvement of 5.4%. By area of residence, almost all urban pregnant women had prenatal consultations during the last pregnancy (95.8% at the end of 2019), while in rural areas, the proportion of rural women whose last pregnancy was monitored by a health specialist reached 85.1%.

In addition, the proportion of women, ages 15-49, who gave birth in a supervised setting improved by nearly 5 percentage points between 2013 and 2019, from 85.4% to 90.8%. This improvement increased from 96.3% to 95.8% in urban areas. In the rural setting, the rate remains well below the national average between 2013 and 2019 (87.4%) although it improved from 72.9% to 85.1% between 2013 and 2019.

#### Theoretical framework for analysis

We rely on the conceptual framework proposed by *Andersen* [28]. This framework provides an analytical tool for identifying and testing causal relationships between access to care and individual and environmental factors [29]. Basing on a philosophical premise, which considers access to health care as a human right, [30, 31] developed an initial behavioral model, which provided a simplified conceptual framework for identifying the determinants of access to medical care and use of health care services in the United States and Canada [30]. This model has been the subject of criticism [32] leading to the gradual, but substantial change of its initial formulation, in particular thanks to the work of *Aday* and *Andersen* [28, 33, 34]. Initially designed around the family as the unit of analysis on the demand side of health care, the model has become more complex through the integration of new dimensions [28]. The current approach differentiates between potential access, which refers more to the supply of available services, and effective access, which refers to the actual use of these services [35], through the introduction of contextual or environmental characteristics into the model.

### Analysis

- Continuous variables were presented as mean and standard deviation, as well as median and range. Absolute frequencies and percentages were used to analyze categorical variables. The association between variables was evaluated using the ANOVA test for continuous variables and the chi-square test for categorical variables.
- All analyses were performed using the R 4.2.1 software

#### Bi-variate analyses of UHC and individual characteristics

To analyze access to medical coverage by different characteristics (socioeconomic and sociodemographic). A sample of 141,067 (2017 wave and 2019 wave) individuals was selected. The table below provides information on the situation of individuals as beneficiaries of the AMO (compulsory health insurance for employees operating in the public and private sector) with high coverage, and of the RAMED with moderate coverage or without medical coverage, broken down according to the different characteristics of the sample.

The analysis of the results shows that overall, 57% of the men in the sample - almost the same as the women in the study (59%) - have medical coverage at a rate of 25% and 24% respectively for the AMO, and almost 32% and 35% for the RAMED, against 43% of men and 41% of women who have no coverage.

The proportions of the different age groups of people with AMO medical coverage are 26% for the age category between 0 and 15 years, 24% for the age category between 15 and 60 years and 29% for those aged 60 years and over. As for the proportions of people with RAMED medical coverage, they are respectively 38%, 31% and 36%. While, those who have no coverage are respectively 36%, 45% and 35%. The geo-spatial analysis of the structure of medical coverage reveals that medical coverage benefits more urban areas (63%) than rural areas (37%), with a predominance of AMO in urban areas (36%) - as opposed to 27% for RAMED - and a predominance of RAMED in rural areas (41%) - as opposed to 12% for AMO, This can be explained by the fact that RAMED is intended for the most disadvantaged social strata, who generally live in rural areas, whereas AMO is a medical coverage intended for employees in the public and private sectors, who mainly live in urban areas. The analysis of the results by marital status reveals that almost half of each category of marital status benefits from medical coverage (i.e. 45% for married people, 53% for single people, and 56% for the rest).

Further reading reveals that married people are the ones who benefit most from AMO medical coverage (29%), followed by single people (22%) and then others (widows, divorcees and others) 17%. For RAMED, widows, divorcees and others benefit the most with 39%, followed by married people with 36%, then singles with 31%. The analysis of the results by level of education confirms that the more educated a person is, the higher the probability that he/she is affiliated to a medical coverage plan.

The structure of beneficiaries of compulsory health insurance (AMO) shows a predominance of people with higher education (52%). This proportion decreases from one level of education to another: 31%, 22% and 17% respectively for people with secondary, primary and no education.

On the other hand, the majority of RAMED beneficiaries are people who have no education (40%). This proportion decreases as the level of education increases (36%, 27% and 11%). The reasoning is the same as for the characteristic “level of education” =  > Same results, 57% of literate people have health insurance, with a predominance for AMO insurance (29%), against 58% for illiterate people with a predominance for RAMED (43%) against AMO (28%). 58% of employed people have medical coverage with a predominance of the proportion of people having AMO (29%) against 23% for those having RAMED. 59% of the unemployed have medical coverage with a preponderance of people having RAMED (34%) against 25% for those having AMO. Households with more than 9 individuals are predominantly those who have no medical coverage. Households with follow this between 5 and 8 people, almost half of whom have no medical coverage, the other half being dominated by RAMED. Households with between 2 and 4 individuals have 60% of medical coverage with almost similar proportions for AMO and RAMED.

For the use of health care services (consultation in case of illness, preventive consultation, prenatal consultation and births assisted by a specialist), two thirds of people who consult in case of illness have health insurance at a rate of 30% for AMO and 37% for RAMED, while 44% of people who do not consult in case of illness, are without any medical coverage. The remaining 56% are dominated by affiliation to the RAMED scheme (37%) against only 19% for the AMO. Almost two thirds of the people who make preventive consultations have a health insurance at the rate of 34% for AMO and 35% for RAMED. The remaining 31% do not have any medical coverage. Of those who did not make a preventive consultation, 57% have health insurance (24% with AMO and 33% with RAMED). 64% of the people who have made prenatal consultations have health insurance, between those affiliated to RAMED (36%) and those affiliated to AMO (28%), against only 57% of the people who have not made prenatal consultations but who have health insurance (11% AMO and 46% RAMED). This result seems a little ambiguous, because if we reason just in terms of beneficiaries of the health insurance scheme, we notice that the proportion of individuals performing prenatal consultations is dominated by RAMED affiliates and we also notice that the proportion of individuals not performing prenatal consultations is also dominated by RAMED affiliates, which means that the fact of performing prenatal consultations is not linked to the nature of the medical scheme to which the person is affiliated. 64% of people, who use specialists for childbirth are covered by health insurance, compared to 46% of people who do not use specialists for childbirth and who have no medical coverage, which may be one of the reasons for not using this type of medical service.

Most people who use public sector health care services have RAMED medical coverage. This seems consistent with the objectives of RAMED. This is in contrast to people who use private sector health care services, most of whom have AMO medical coverage.

Among the chronically ill, 38% have RAMED-type medical coverage, 34% have no medical coverage, and 28% have AMO medical coverage. Amid those in good health, 43% have no medical coverage. The income groups that benefit the most from AMO insurance are the wealthiest, unlike RAMED, whose beneficiaries are the poorest. It should be noted that as income increases, the probability that an individual will be affiliated to AMO increases to the detriment of RMAED or of not being affiliated to any health plan.

In the same stream, the regions with the highest number of people receiving AMO are the regions with the highest incomes, namely the southern regions (*Dakhala Oued* D*a*ℎ*ab*, *Sakia* A*l* H*amra* and *Guelmim* O*ued* N*o*u*n*), followed by the metropolis of Casablanca and the region of *Rabat* − *Sal*é – *K*é*nitra* where there is a strong concentration of capital.

The main region with the highest number of people benefiting from RAMED is the Eastern region (50%). As for the northern region of the kingdom (*Tang*i*er* − *T*e*touan* − *Houceima*) presents the highest number of people who have no medical coverage, that is 54%.

It is important to note that the frustration related to geographical location is even more present among the inhabitants of Tetouan and Beni Mellal, as they are often referred to other cities for care. The residents of Tetouan opt for Tangier, Rabat and Casablanca in that order, as the costs increase proportionally with the distance. For the same reason, the inhabitants of Beni Mellal often go to Marrakech or Casablanca and rarely to Rabat. Nevertheless, regardless of the destination, travelling requires significant costs for transportation, food and sometimes accommodation.

#### Bivariate analyses of access to health services and individual characteristics

The analysis of the result shows that the proportion of women who sought health care services is slightly higher than that of men. As for age, the elderly has more access to health care services than the other age categories. With regard to geo-spatial dispatching, the results show that people who live in urban areas have easier access to health care services than people who live in rural areas. This can be explained by the proximity of health centers in urban areas than in rural areas, the availability of medical staff and health infrastructures in urban areas than in rural areas.

The analysis of educational attainment for accessing health care reveals that the more educated a person is the more health care they access. This seems logical and consistent since access to health care services requires that the individual should have a certain level of awareness, which is positively correlated with the level of education. The results of the “literacy” characteristic support this finding.

The analysis of the characteristic “employment” shows that having a job can increase the probability to access to health care. As for the size of the household, it presents a mixed result that does not leave any conclusion.

People with chronic illnesses conditions use health care services more than healthy people.

People with AMO medical coverage are more likely to access the various health care services than people subject to RAMED and even less likely for people with no medical coverage.

The analysis of access to health care by income class shows that the higher the income, the greater the access to health care services. This may be explained by the fragility of the health system and public health policies, by high health care costs, or by individuals' personal choices regarding to health care spending.

With regard to the analysis by region, the regions with the highest number of people with access to health care are the southern regions, namely *Dak*ℎ*la Oued Edda*ℎ*ab*, *Guelmim* Oued *Noun* and *Laayoun Sakia Hamra* followed by the metropolis of Casablanca and the region of *Rabat Sal*é *K*é*nitra* region, at a lower proportion. On the other hand, the regions with the highest proportion of subjects who have not made a medical consultation after having suffered from an illness are *Tanger Tetouane Al* H*ouceima* and *Beni* M*elal K*ℎe*nifra*.

The geographic location is a factor to consider when analyzing access to health services particularly for the rural population. The results of our qualitative and quantitative study showed that the perception of the overall cost varies according to the region and the Proximity to health services. Thus, different difficulties are encountered by the population depending on the area of residence.

#### Model specification

The descriptive bivariate results showed the extent to which demographic, socioeconomic, and geographic factors are related to actual use of health services and to the type of medical coverage (AMO and RAMED).

We tested different models by using the multiple logistic regression technique in order to verify the contribution of each factor in the use of health services, taking into consideration other determinants. The equation models are in the form of regression coefficients related to each variable of interest in the generalized linear model (logistic regression) and the interpretation of each factor uses *odds ratio* (OR) measured by the exponential of the regression coefficients and their 95% confidence intervals.

#### The logit model uses a logistic function as density function

In what follows, we write the form of the post-estimated regression model.

Let us posit:

$${\varvec{P}}({\varvec{Y}}=1|\boldsymbol{ }{\varvec{X}}1,\boldsymbol{ }{\varvec{X}}2,\boldsymbol{ }\dots ,\boldsymbol{ }{\varvec{X}}{\varvec{k}})=\boldsymbol{ }{\varvec{G}}({\varvec{X}}\boldsymbol{^{\prime}}{\varvec{\beta}})$$

With G is a distribution function (non-linear with respect to the parameters of the model and between 0 and 1).

Indeed, in the case of a *Logit* model:

$${\varvec{P}}\left({\varvec{Y}}=1\right)={\varvec{G}}\left({\varvec{X}}{\varvec{\beta}}\right)=\frac{{{\varvec{e}}}^{{\varvec{X}}{\varvec{\beta}}}}{1+{{\varvec{e}}}^{{\varvec{X}}{\varvec{\beta}}}}$$

The model was estimated using the maximum likelihood method, and the results are presented in terms of marginal effects.

#### Discussion of results

The question now is to interpret the impact of the selected demographic and socioeconomic characteristics on the use of medical care. The estimation of the econometric model adopted, shows that medical coverage with these two components (AMO and RAMED*)* has a positive and significant impact on the probability of using health care services (*p* < 0.05). In fact, people who have medical coverage are more likely to seek out a specialist than those who have no coverage. This proves that the implementation of universal health coverage would allow, or even encourage, individuals, particularly beneficiaries, to seek out healthcare specialists during the event of illness.

This result is perfectly in line with the conclusions of previous works [9, 11, 12, 36]. In addition to the influence of health coverage, the impact of demographic, socio-economic and socio-cultural characteristics is confirmed with a significant difference between, on the one hand, rich females with a higher level of education, having a job and living in metropolitan areas, especially in urban areas, and, on the other hand, poor males’ subjects with a lower level of education, not having a job, coming from a large family and living in peripheral areas. In more detail, we find that demographic and socioeconomic factors such as gender, age, level of education, standard of living (approximated by annual household expenditure), place of residence, etc., play an important and statistically significant role in seeking health care services.

Indeed, people who live in urban areas are more likely to access a health care provider than those who live in rural areas (OR = 1.39 in 2017 highly significant at the 1% risk level and OR = 1.08 in 2019 but not significant at the 5% level). This is mainly due to the absence of health centers in some enclaves or, in the best cases, to the presence of difficulties in accessing health infrastructure.

This finding affirms the conclusions raised by the economists [11, 37, 38] who have shown the importance of the residential environment in the context of health services use. Therefore, we can confirm that people, depending on whether they belong to urban or rural areas, present different perceptions of the use of health services.

As far as gender is concerned, women consult more than men. Indeed, the probability of using health care services increases when it is the female gender (OR = 1.37 in 2017 and OR = 1.25 in 2019). The differential between the two genders can be explained by women's vulnerability to illnesses caused, on the one hand, by the increase in childbirth and, on the other, by the burden of responsibilities and domestic work. This result which is consistent with the work of the economists [39] assumes that women are the most willing to use health services compared to men. In contrast, there is no significant difference between age categories in terms of access to care for either the 2017 wave, or the 2019 wave.

Regarding to health status, having at least one chronic disease increases the probability of visiting a specialist twice as much as someone without a chronic disease (OR = 2.2 in 2017 and OR = 2.21 in 2019 with a risk threshold below 1%). At the same time, the model’s results reveal the importance of other socioeconomic determinants in relation to whether or not people forego healthcare.

The probability of using health care services increases gradually with the increase in the individual's standard of living (1^st^ quintile, 2^nd^ quintile, 3^rd^ quintile, and 4^th^ quintile). Thus, being richer increases the chance of consulting a health care specialist: in fact, the richer a person is, the more likely he or she is to use health care. People who belong to the second quintile have (OR = 1.36) 1.36 times more chance than People who belong to the first quintile. Similarly, subjects belonging to the third quintile have (OR = 2.03) 2.03 times more chance than people who belong to the 1st quintile and people who belong to the fourth quintile have (OR = 3.01) 3 times more chance than subjects belonging to the first quintile. These results hold for both years (2017 and 2019) and are highly significant at even the 1% level. On the other hand, the level of education remains insignificant, which contradicts the model of Grossman [10], that confirms the extent and the primordial role that education level can play in determining the health behavior of individuals.

## Conclusion

This work has highlighted several important findings. The study identifies demographic and socioeconomic factors as the main determinants of access to health care services, including possession of health insurance, place of residence, gender, and standard of living. Through quantitative analysis of longitudinal data, the study reveals a significant difference in the utilization of health care services between people covered and those who are not covered by any type of insurance. Indeed, the members who affiliate a mingled type of medical coverage consult health care providers as well as their descendants more than the people without any coverage. These results support the findings of previous theoretical [10] and empirical studies [11]. The primary inference drawn from the study's findings is that the widespread implementation of health coverage would extend access to previously unattainable medical services, specifically treatments for conditions like renal failure, hepatitis, and cardiovascular diseases, particularly benefiting individuals facing economic disadvantages.

## Recommendation

To promote public health in Morocco, it is imperative to cultivate a culture of health consciousness through awareness campaigns and elevate the caliber of healthcare services. The Ministry of Health should embark on thorough reforms, modernizing the sector to meet the changing needs and expectations of the population. This involves ensuring both the quality and equity of healthcare delivery.

## Data Availability

The datasets generated and analyzed during the current study are available in the ONDH data repository on reasonable request https://www.ondh.ma/fr/data-ondh.

## Abbreviations

UHC: Universal Health Coverage
RAMED: Régime d’Assistance Médical aux Economiquement Démunis
AMO: Assurance Maladie Obligatoire
OOP: Out Of Pocket
HPS: Household Panel Survey
ONDH: National Observatory of Human Development
